# Zirconia Nanoparticles-Induced Toxic Effects in Osteoblast-Like 3T3-E1 Cells

**DOI:** 10.1186/s11671-018-2747-3

**Published:** 2018-11-06

**Authors:** Mingfu Ye, Bin Shi

**Affiliations:** 1 The State Key Laboratory Breeding Base of Basic Science of Stomatology (Hubei-MOST) and Key Laboratory for Oral Biomedical Ministry of Education, School and Hospital of Stomatology, Wuhan University, Wuhan, People’s Republic of China; 2 Department of Implantology, Xiamen Stomatology Hospital, Hospital and School of Stomatology, Xiamen Medical University, Xiamen, 361003 People’s Republic of China; 3 Department of Implantology, School and Hospital of Stomatology, Wuhan University, 237 Luoyu Rd, Wuhan, 430072 People’s Republic of China

**Keywords:** Zirconia, Titanium dioxide, Nanoparticles, 3T3-E1 cells, Cytotoxicity, Osteogenesis

## Abstract

Zirconia (ZrO_2_) is one of the widely used metal oxides for potential bio-applications such as biosensors, cancer therapy, implants, and dentistry due to its high mechanical strength and less toxicity. Because of their widespread applications, the potential exposure to these nanoparticles (NPs) has increased, which has attracted extensive attention. Thus, it is urgent to investigate the toxicological profile of ZrO_2_ NPs. Titanium dioxide (TiO_2_) is another extensively used nanomaterials which are known to be weakly toxic. In this study, TiO_2_ NPs were served as control to evaluate the biocompatibility of ZrO_2_ NPs. We detected the cytotoxicity of TiO_2_ and ZrO_2_ NPs in osteoblast-like 3T3-E1 cells and found that reactive oxygen species (ROS) played a crucial role in the TiO_2_ and ZrO_2_ NP-induced cytotoxicity with concentration-dependent manner. We also showed TiO_2_ and ZrO_2_ NPs could induce apoptosis and morphology changes after culturing with 3T3-E1 cells at high concentrations. Moreover, TiO_2_ and ZrO_2_ NPs at high concentrations could inhibit cell osteogenic differentiation, compared to those at low concentrations. In conclusion, TiO_2_ and ZrO_2_ NPs could induce cytotoxic responses in vitro in a concentration-dependent manner, which may also affect osteogenesis; ZrO_2_ NPs showed more potent toxic effects than TiO_2_ NPs.

## Introduction

During the past few decades, the application of engineered nanoparticles (NPs) has expanded in various fields, such as electronics, biomedical applications, and pharmaceuticals. Zirconia (ZrO_2_) NPs are one of the major nanomaterials used for synthesizing refractories, foundry sands, and ceramics. Due to the preferable mechanical strength, this material is also used in biomedical field, including biosensors, cancer therapy, implants, joint endoprostheses, and dentistry [1, 2]. However, the wide application of particles has raised concern on their potential risks to health and environment, of which ensuring occupational and consumer safety is an essential concern. So far, toxicological studies on ZrO_2_ NPs are limited, and the results were controversial.

Some studies have reported that ZrO_2_ NPs showed better biocompatibility when compared with other nanomaterials, including ferric oxide, titanium dioxide (TiO_2_), and zinc oxide (ZnO) [3–6]. In agreement with these results, others have reported ZrO_2_ NP could induce mild [3, 7] or no cytotoxic effects [8–10], and only few studies indicated a mild cytotoxic potential. However, Stoccoro et al. [11] developed the toxic effects of ZrO_2_ NPs and TiO_2_ NPs coated or not, they found that all kinds of NPs showed toxic effects to different degrees. Moreover, cell morphology changes, and cracks on the cell surface were observed in another study after ZrO_2_ NP treatment at concentrations up to 1 mg/mL in the red blood cells [12]. Hence, in this study, we evaluated the cytotoxic effects of ZrO_2_ NPs, providing useful insight for their future application in vivo. Meanwhile, we treated the cells with TiO_2_ NPs as the control group, which toxicological profile has been well developed [13].

Previous studies showed that NPs have been widely used as tissue-engineered materials and have the ability to improve the osteogenic differentiation of osteoblast [14–17]. One report indicated that silica (Si) NPs could reverse age-associated bone loss in mice, probably due to the Si NP-induced bone formation [16]. Liu et al. [14] found that silver (Ag) NPs/poly (DL-lactic-co-glycolic acid)-coated stainless steel alloy has strong antibacterial ability and could promote MC3T3-E1 cells osteoblastic proliferation and maturation in vitro. Moreover, carbon nanotubes were reported to induce bone calcification, most likely result from their nanosized structures which are similar to the size of intracellular organelles [18].

ZrO_2_ NPs have been applied as the main component of bioceramic implants, owing to its biocompatibility and resistance to bio-corrosion [19]. Although majority of studies have focused on the advantageous properties of ZrO_2_ NPs, the adverse biological effects are impossible to be neglected. Therefore, in this study, we used TiO_2_, as a control group, which is a traditional nanomaterial that showed similar physicochemical properties. We aimed to investigate the effects of TiO_2_ and ZrO_2_ NPs on cell viability, oxidative stress, cell morphology, and osteogenic responses of MC3T3-E1 osteoblasts after co-culture and thus to reveal the osteoinductivity of TiO_2_ and ZrO_2_ NP treatment.

## Materials and Methods

### Materials Preparation and Characterization

TiO_2_ NPs (CAS Number 637262) and ZrO_2_ NPs (CAS Number 544760) were purchased from Sigma-Aldrich (Sigma-Aldrich, St. Louis, MO, USA) and characterized by transmission electron microscope (TEM, MFP-3D-S, Asylum Research, Santa Barbara, CA, USA), Zeta potential, and dynamic light scattering (DLS) particle size analysis measurements (Zetasizer Nano ZS, Malvern, UK). The NPs were dispersed in alcohol for TEM detection, which could show the morphology and particulate size of NPs more clearly. In addition, the aggregated size of the particles was detected via DLS, where complete culture medium was used to bring into correspondence with particle characters applied in cell culture. Before cell treatment, the stock solution was dispersed by Ultrasonic Cell Disruption System (Ningbo Xinzhi Biotechnology, China) for 30 min accompanied with ice cooling and diluted to different concentrations with complete culture medium prior to cell experiments.

### 3T3-E1 Cell Culture

3T3-E1 cell line (the Cell Bank of the Shanghai Infrastructure for Public Research and Development of the Chinese Academy of Medical Sciences, Shanghai, China) was cultured in minimum essential medium-alpha (α-MEM, Thermo Fisher Scientific, Waltham, MA, USA) containing 10% fetal bovine serum (FBS, Thermo Fisher Scientific, USA) and 1% antibiotic/antimycotic (Thermo Fisher Scientific, USA). Cells were incubated at 37 °C with 5% CO_2_ in a 95% humidified atmosphere, and the culture medium was replaced every other day.

### Cell Proliferation Assay

Cellular viability was detected using the CCK-8 assay (Dojindo Molecular Technologies, Kumamoto city, Japan). Cells were seeded in 96-well plates at 5000 cells per well. TiO_2_ NPs and ZrO_2_ NPs were then added to the 96-well plates at serial concentrations of 0, 10, 20, 40, 60, 80, 100, and 150 μg/mL followed by incubation for 24 and 48 h at 37 °C with 5% CO_2_, accompanied with *N*-acetyl-l-cysteine (NAC) or not, which were used to inhibit ROS production. The control group was left untreated. Then, the CCK-8 test was conducted by adding 110 μL detection reagents to each well, and the 96-well plates were then incubated for an additional 2 h at 37 °C. To prevent NPs from interfering in this analytical assay, the reagents to be tested in the 96-well plates were transferred to a new 96-well plate after 2 h reaction time; the deposited NPs and cells were left in the primary plate. The optical density (OD) of each well was measured at a single wavelength of 450 nm with the microplate reader (SpectraMax M5, Molecular Devices, Sunnyvale, CA, USA). Each treatment was done in six replicates.

### Annexin V Apoptosis Analysis by Flow Cytometry

Cells were cultured in a 12-well plate at a density of 30,000 cells/well for confluency. After TiO_2_ NP and ZrO_2_ NP treatment for 48 h, cells were washed with PBS and collected using EDTA free-trypsin buffer. Cells were resuspended with PBS buffer at a concentration of 25,000 cells/mL and centrifuged at 1000×*g*. Then, cells were stained with FITC Annexin V and PI (Invitrogen™, USA) at room temperature without light exposure. Finally, cells were mixed with 400 μL of binding buffer and analyzed immediately by flow cytometry (BD FACSAria III, BD, Franklin Lakes, NJ, USA).

### ROS Generation Analysis

The formation of intracellular ROS was determined using the Reactive Oxygen Species Assay Kit (Beyotime, Shanghai, China). Briefly, after washing with PBS, cells were seeded in a 6-well plate at 20,000 cells/well in 2 mL culture medium and treated with TiO_2_ NPs and ZrO_2_ NPs at a concentration of 0, 10, 50, and 100 μg/mL for 48 h, accompanied with NAC or not. After treatment with TiO_2_ NPs and ZrO_2_ NPs, cells were collected and incubated with 10 μM DCFH-DA for 30 min at 37 °C and 5% CO_2_. Fluorescent intensities were analyzed on a BD FACSAria III (BD, Franklin Lakes, NJ, USA).

### Confocal Microscopy

Due to a large proportion of cells have turned into apoptosis status, we chose 24 h as the time point to observe the cells’ cytoskeleton structure changes in our study. 3T3-E1 cells were seeded on glass coverslips and cultured in the presence of TiO_2_ NPs and ZrO_2_ NPs for 24 h. Cells were washed immediately after the treatment with PBS buffer for three times and fixed with 4% paraformaldehyde, permeabilizated with 0.1% Triton X-100, and blocked with PBS containing 5% BSA. Then, cells were incubated for α-tubulin (Sigma-Aldrich, St. Louis, MO, USA, 1:4000) at 4 °C overnight and loaded with the FITC-bound secondary antibody at 37 °C for 1 h next day after washing with PBS for three times. Consequently, cytoskeleton was stained by rhodamine-phalloidin (Invitrogen, Carlsbad, CA, USA, 1:1000) for 1 h in dark, and the nuclei were stained with Hoechst 33342 (Invitrogen, Carlsbad, CA, USA) for 20 min. Coverslips were examined using a FV10i confocal microscope (Olympus, Tokyo, Japan).

### Mineralization Induction Detection

3T3-E1 cells were seeded in a 6-well plate at a density of 15,000 cells/well. The cells were treated with TiO_2_ NPs and ZrO_2_ NPs at concentrations of 10 and 100 μg/mL, and the culture medium containing the nanomaterials was replaced every other day; the cells were washed gently via PBS to remove the residual nanomaterials before every culture medium changes. After culturing for 7, 14, and 21 days in the presence of TiO_2_ NPs and ZrO_2_ NPs, the cells were stained by alizarin red S. Briefly, cells were fixed with 4% paraformaldehyde at 4 °C for 30 min and stained with alizarin red S solution (40 mM, pH 4.1) at ambient temperature for another 20 min. After washing with distilled water three times, mineralized nodules were observed with a light microscope (Olympus, Japan).

### RNA Extraction and RT-PCR

Total RNA was extracted via TRIzol reagent (Invitrogen, Carlsbad, CA, USA). Then, RNA concentration was evaluated using an ultraviolet spectrophotometer. The isolated RNA was reverse transcribed to cDNA using a RT reagent kit (TaKaRa Bio, Dalian, China). Real-time PCR was carried out using SYBR green reagent (TaKaRa Bio, Dalian, China). Osteogenesis-related genes were detected, including runt-related transcription factor 2 (RUNX2), collagen 1α1 (Col1α1), alkaline phosphatase (ALP), osteopontin (OPN), osteocalcin (OC), and bone sialoprotein (BSP). The data were analyzed using the 2^−ΔΔ^CT method. The primers used were listed in Table 1.

**Table 1:** The primers list for RT-PCR

Gene	Forward primers	Reverse primers
*β-actin*	TACAGCTTCACCACCACAGC	TCTCCAGGGAGGAAGAGGAT
*Runx2*	ACAGTCCCAACTTCCTGTGC	ACGGTAACCACAGTCCCATC
*Col1α1*	ACGTCCTGGTGAAGTTGGTC	TCCAGCAATACCCTGAGGTC
*ALP*	ACAACCTGACTGACCCTTCG	TCATGATGTCCGTGGTCAAT
*OPN*	CACCATTCGGATGAGTCTGA	CCTCAGTCCATAAGCCAAGC
*OC*	GAGGACCCTCTCTCTGCTCA	ACCTTATTGCCCTCCTGCTT
*BSP*	TGTCCTTCTGAACGGGTTTC	TCGTTGCCTGTTTGTTCGTA

### Statistical Analysis

The results were represented as the means ± SEM. All data were statistically analyzed by ANOVA test. The homogeneity of variance test was performed, and Bonferroni and Dunnett’s T3 tests were used when the equal variance was assumed and when there was no homogeneity, respectively. *p* value less than 0.05 was considered statistically significant.

## Results

### Characterization of the TiO_2_ and ZrO_2_ NPs

**Fig. 1:**
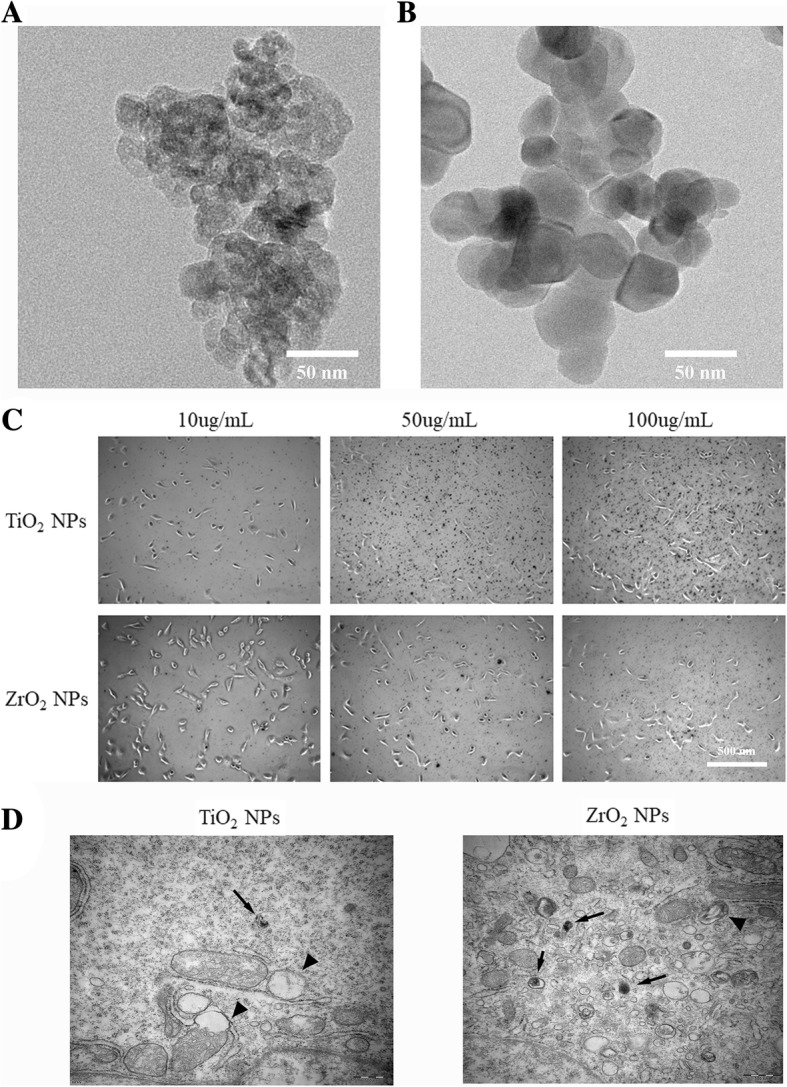
Characterizations of the TiO_2_ and ZrO_2_ NPs. TiO_2_ (**a**) and ZrO_2_ (**b**) NP morphology and size were detected using TEM. (**c**) The co-culture situation of 3T3 cells and nanomaterials was observed after TiO_2_ and ZrO_2_ NP treatment concentrations of 10, 50, and 100 μg/mL. (**d**) The TEM results were obtained after TiO_2_ and ZrO_2_ NP treatment for 1 h

We first characterized the TiO_2_ NP and ZrO_2_ NP powders via transmission electron microscopy (TEM) and dynamic light scattering (DLS) (Fig. 1a, b, Table 2). The TEM and SEM images revealed the particle shapes and sizes. The TiO_2_ NPs were small rod-shaped spheres with an average size of 25.4 ± 2.8 nm. The ZrO_2_ NPs were small rod-shaped spheres with an average size of 31.9 ± 1.9 nm. To measure the size of TiO_2_ NPs and ZrO_2_ NPs in solution, DLS was used and the particles of TiO_2_ NPs and ZrO_2_ NPs expanded to 81.2 nm and 93.1 nm, respectively, which indicated an agglomeration effect. The zeta potentials of TiO_2_ NPs and ZrO_2_ NPs were 32.9 ± 5.4 mV and 42.4 ± 7.4 mV, respectively.

**Table 2:** Characterization of the TiO_2_ andZrO_2_ NPs

Nanoparticles	Average size (nm)	DLS (nm)	Zeta potential (mV)
TiO_2_	25.4 ± 2.8	81.2	32.9 ± 5.4
ZrO_2_	31.9 ± 1.9	93.1	42.4 ± 7.4

Then, we observed the photograph of 3T3 cells after TiO_2_ NP and ZrO_2_ NP exposure at various concentrations. We found that the NPs distributed evenly on the cells or spread around. The NPs showed potent aggregation ability at high concentrations due to a small fraction of NPs with microscale observed, while a great mass of NPs was small with nanoscale and probably translocates into cells which was hard to see (Fig. 1c). Furthermore, TEM results of cells after TiO_2_ and ZrO_2_ NP treatment for 1 h have been obtained; our data showed that NPs could be translocated into cellular vesicles. Meanwhile, some organelle damages are also observed, for example, mitochondrial swell and vacuole occurred.

### TiO_2_ and ZrO_2_ NP-Induced Toxic Effects in 3T3-E1 Cell

**Fig. 2:**
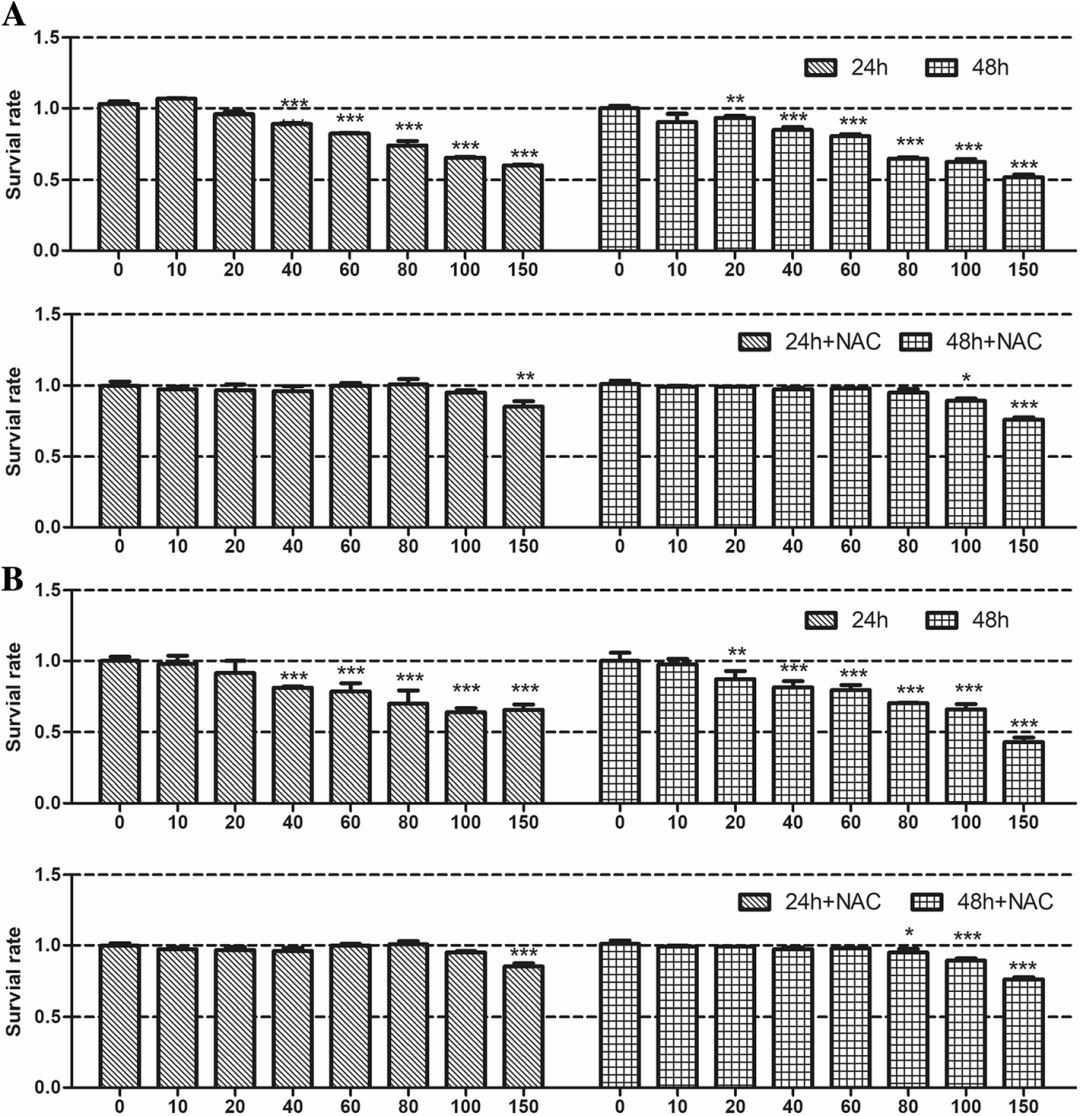
TiO_2_ and ZrO_2_ NP-induced cell viability decrease in 3T3-E1 cells. 3T3-E1 cells were treated with TiO_2_ (**a**) and ZrO_2_ (**b**) NPs at concentrations of 0, 10, 20, 40, 60, 80, 100, and 150 μg/mL for 24 and 48 h, and then the cell viability was detected via the CCK-8 assay. Meanwhile, the cell viability changes were detected after NAC treatment, which could eliminate intracellular ROS. The results represent the means ± SEM. *p < 0.05; **p < 0.01; ***p < 0.001, compared with the control

We assessed cell viability after TiO_2_ NP and ZrO_2_ NP treatment in series concentrations (10, 20, 40, 60, 80, 100, 150 μg/mL). For TiO_2_ NPs (Fig. 2a), after 24 h of incubation, we found that TiO_2_ NPs were non-toxic at lower doses (≤ 20 μg/mL), whereas an obvious decrease in cell viability was observed at higher concentrations (> 20 μg/mL) (*p* < 0.001). More dramatic decrease of cell viability in the 20 μg/mL treatment group was observed after 48 h of incubation; TiO_2_ NPs at the concentration of 20 μg/mL induced cell viability decrease (*p* < 0.01). In addition, higher doses of TiO_2_ NPs (> 20 μg/mL) showed significant decrease of cell viability at 48 h (*p* < 0.001). However, cell viability remained stable when treated with 10 μg/mL of TiO_2_ NPs for 48 h. Moreover, for ZrO_2_ NPs (Fig. 2b), similar results were observed when compared with TiO_2_ NPs; higher toxic effects were observed at the concentration of 150 μg/mL for 48 h, where cell viability decrease below 50%. These results indicated that TiO_2_ and ZrO_2_ NPs were biocompatible at lower doses. However, these two nanomaterials showed slight cytotoxicity at high toxic concentrations.

### NAC Inhibition Effects on the TiO_2_ and ZrO_2_ NPs-Induced Cytotoxicity

We then detected the inhibition effects of NAC which is an ROS scavenging agent. The results showed that NAC potentially inhibited TiO_2_ (Fig. 2a), and ZrO_2_ NPs (Fig. 2b) induced cell viability after 24 h and 48 h treatment. After NAC inhibition, cell viability was maintained for 24 h at all concentrations of TiO_2_ and ZrO_2_ NP treatment except the highest concentration (150 μg/mL). Although the inhibition effect was slightly decreased in cells treated with high concentrations of TiO_2_ (100 and 150 μg/mL) and ZrO_2_ NPs (80, 100 and 150 μg/mL) at 48 h time point, no potent cell viability changes were observed for concentrations below 80 μg/mL, where cell viability was significantly higher than those without NAC.

### TiO_2_ and ZrO_2_ NPs-Induced ROS Generation in 3T3-E1 Cell

**Fig. 3:**
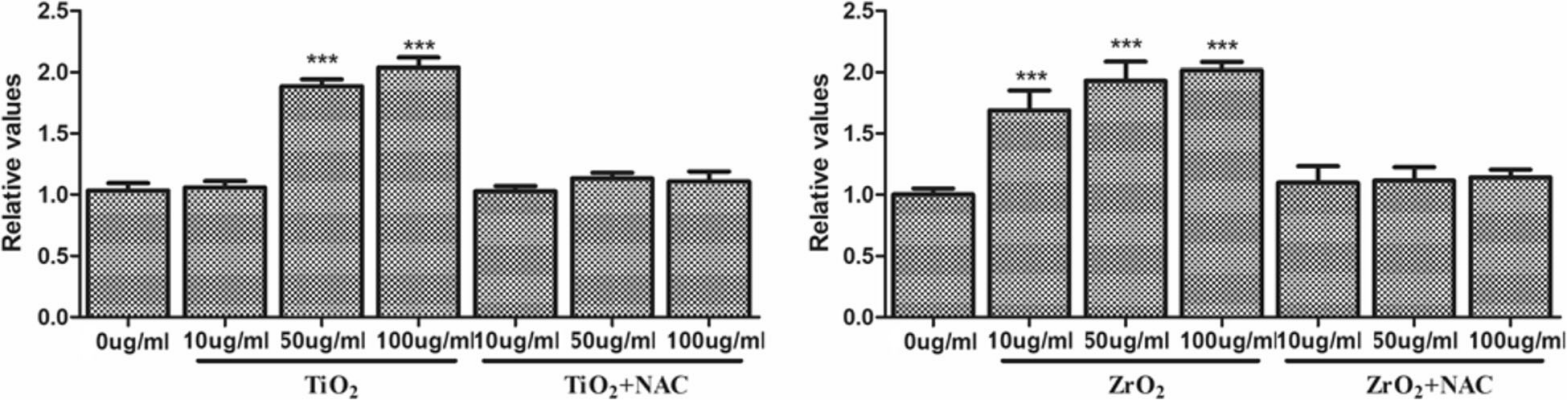
TiO_2_ and ZrO_2_ NP-induced ROS generation in 3T3-E1 cells. 3T3-E1 cells were treated with TiO_2_ and ZrO_2_ NPs at various concentrations for 48 h, and NAC (10 mM) was incubated simultaneously, and then the ROS levels in the 3T3-E1 cells were detected. The results represent the means ± SEM. *p < 0.05; **p < 0.01; ***p < 0.001, compared with the control

We further detected the ROS generation after TiO_2_ and ZrO_2_ NPs exposure in 3T3-E1 cells (Fig. 3). Our results showed that TiO_2_ and ZrO_2_ NPs induced ROS generation after 24 h, which was the most significant at the concentration of 100 μg/mL. There was no significant ROS generation for TiO_2_ NPs at a concentration of 10 μg/mL, while ZrO_2_ NPs induced potent ROS generation at the same concentration. Meanwhile, NAC could significantly inhibit TiO_2_ and ZrO_2_ NP-induced ROS generation in 3T3-E1 cell at all concentrations.

### TiO_2_ and ZrO_2_ NPs-Induced Apoptosis and Necrosis in 3T3-E1 Cell

**Fig. 4:**
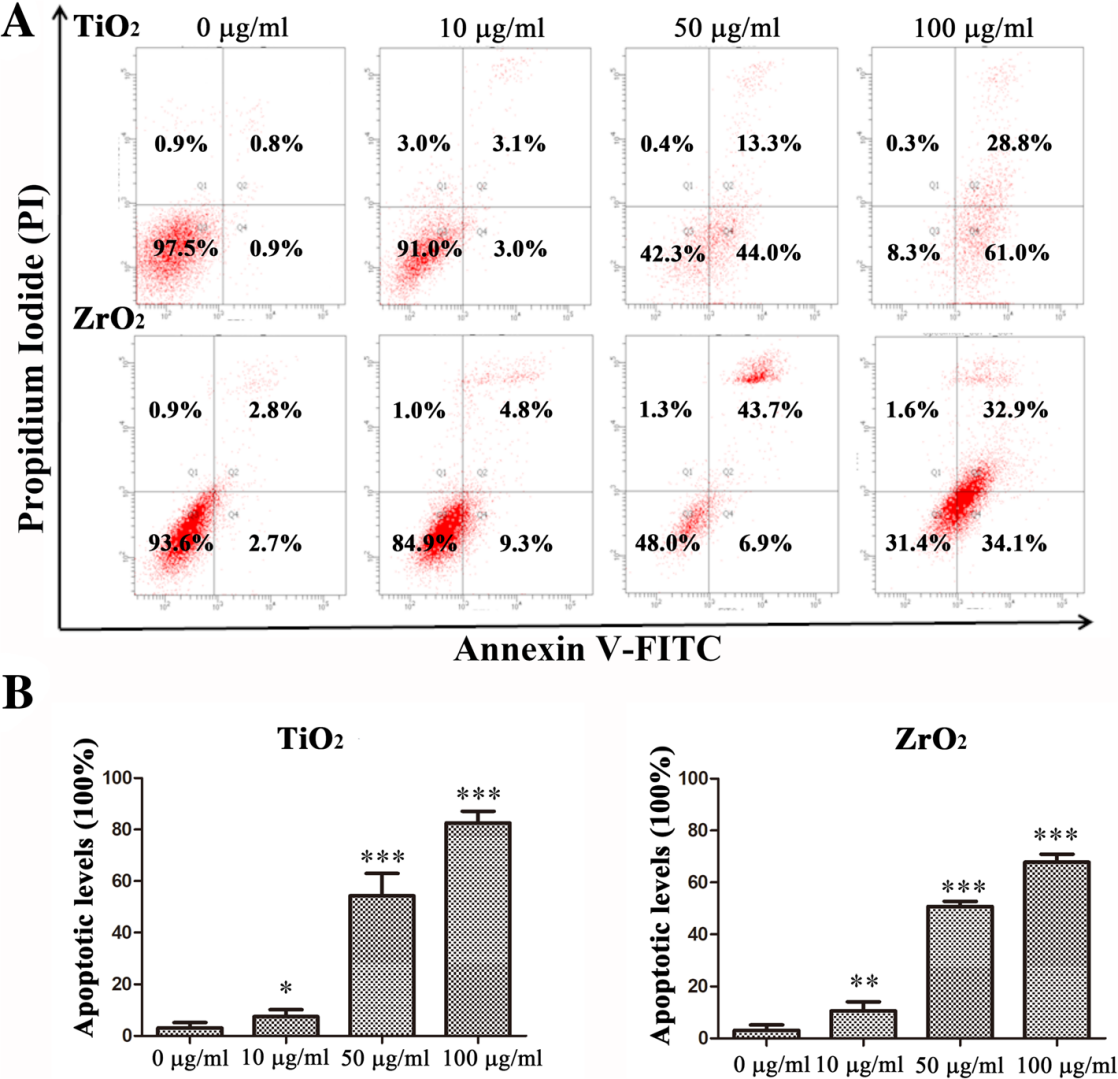
TiO_2_ and ZrO_2_ NP-induced apoptosis in 3T3-E1 cells. **a** After the 3T3-E1 cells were treated with the TiO_2_ and ZrO_2_ NPs at various concentrations for 48 h, the levels of cell apoptosis were detected. **b** The apoptotic levels including early apoptosis and late apoptosis levels were calculated, and then the data carried out the statistics. The results represent the means ± SEM. *p < 0.05; **p < 0.01; ***p < 0.001, compared with the control

Cell apoptosis and necrosis were detected after various concentrations of TiO_2_ and ZrO_2_ NP exposure at 48 h (Fig. 4). The red dots located in the third quadrant represented normal cells, while the red dots located in the first quadrant and fourth quadrant represented the early apoptotic cells and late apoptotic or necrotic cells, respectively. Interestingly, our results indicated that TiO_2_ and ZrO_2_ NPs could induce apoptosis in concentration- and time-dependent manners. Following the TiO_2_ NP exposure for 48 h, no significant cell apoptosis was detected at concentrations of 10 μg/mL; however, at concentrations of 50 and 100 μg/mL, the percentage of late apoptotic or necrotic cells reached to high levels. Following the ZrO_2_ NP exposure for 48 h, we did not find cell apoptosis at the concentration of 10 μg/mL too, but the percentage of late apoptotic or necrotic cells was 43.7% at 50 μg/mL group. Most interestingly, significant early apoptosis was observed (34.1%) at the concentration of 100 μg/mL after 48 h treatment. We found that the early apoptosis levels of TiO_2_ NPs were significantly higher than ZrO_2_ NPs; however, the late apoptotic or necrotic levels were in verse.

### TiO_2_ and ZrO_2_ NP-Induced Morphological Changes in 3T3-E1 Cells

**Fig. 5:**
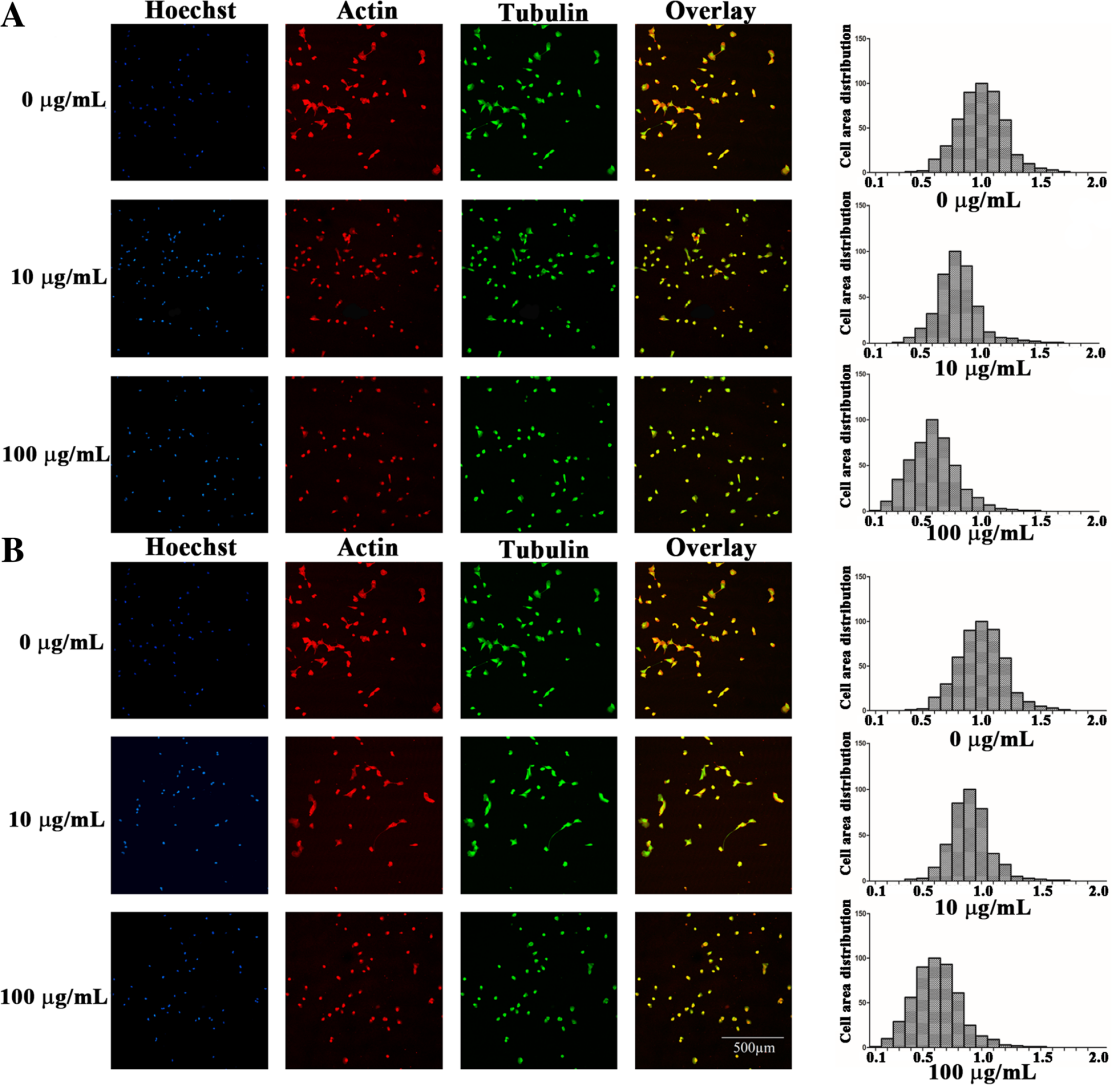
TiO_2_ and ZrO_2_ NP-induced cell area changes in 3T3-E1 cells. After the 3T3-E1 cells were treated with the TiO_2_ (**a**) and ZrO_2_ (**b**) NPs at concentrations of 10 and 100 μg/mL for 24 h, the cells were loaded with tubulin (green), actin (red), and Hoechst 33342 (blue). The cell morphology was observed based on the alterations of the actin (red) and tubulin systems (green), and the cell area distribution changes were calculated

**Fig. 6:**
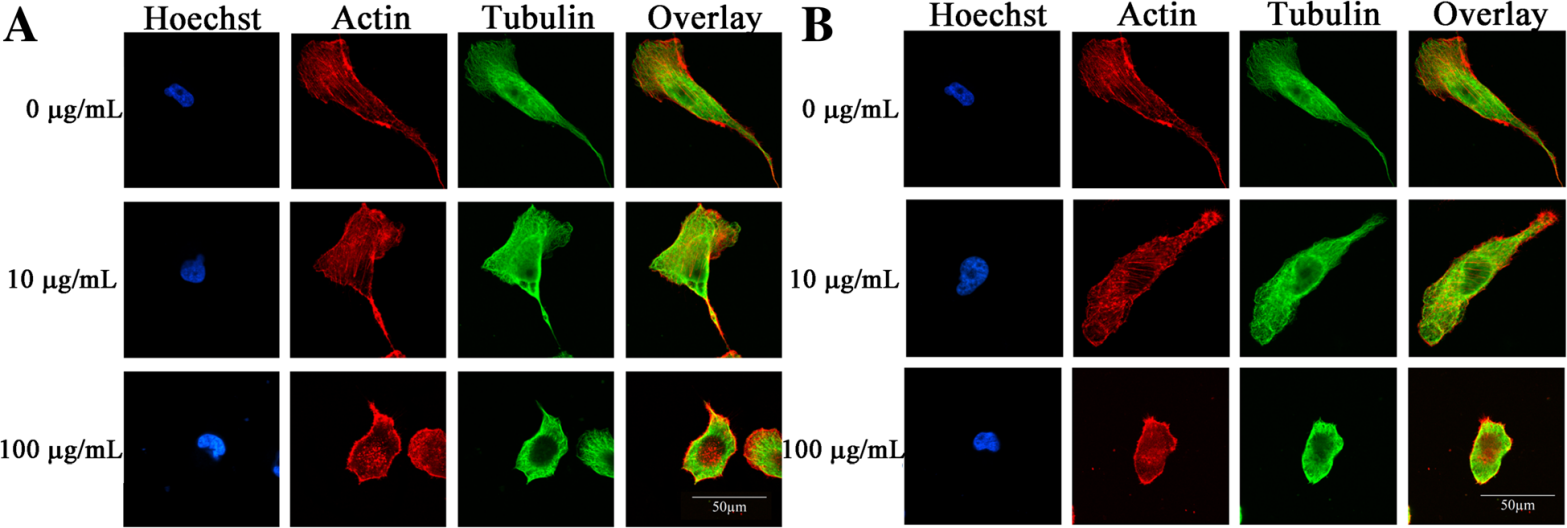
TiO_2_ and ZrO_2_ NP-induced cytoskeleton changes in 3T3 cells. After the 3T3-E1 cells were treated with the TiO_2_ (**a**) and ZrO_2_ (**b**) NPs at concentrations of 10 and 100 μg/mL for 24 h, the cytoskeleton changes were assessed based on the alterations of the actin (red) and tubulin systems (green)

To study the morphological changes of 3T3-E1 cells after exposure to TiO_2_ and ZrO_2_ NPs, we performed fluorescence staining followed by confocal microscopy (Figs. 5 and 6). Compared with untreated control cells, there was no morphological change after 10 μg/mL of TiO_2_ and ZrO_2_ NP treatment at 24 h, while cells turned to become round and smaller after 100 μg/mL of TiO_2_ and ZrO_2_ NP treatment. Most interestingly, slight cell area decrease was observed after 50 μg/mL of TiO_2_ and ZrO_2_ NP treatment, where TiO_2_ showed more potent cell area decrease. Consistently, quantitative results confirmed a significant decrease in cell area after 100 μg/mL of TiO_2_ and ZrO_2_ NP treatment (Fig. 5).

We further studied the cytoskeleton changes in both actin filaments and microtubule levels (Fig. 6). Similarly, no significant difference was shown between control group and 10 μg/mL of TiO_2_ and ZrO_2_ NP treatment, and cells in both groups revealed explicit structures of actin filaments and microtubule system. In contrast, 100 μg/mL of ZrO_2_ NP treatment induced a shrinkage of 3T3-E1 cells, along with a pyknosis-like nuclei and condensed unclear actin filaments and microtubule structures. For TiO_2_ NPs, so many actin dots were observed, and the actin filaments located at the cell membrane were misty and rough. For ZrO_2_ NPs, more potent cytoskeleton disruption was detected, and actin and microtubule structure were rough and defective.

### TiO_2_ and ZrO_2_ NP-Induced Mineralization in 3T3 Cells

**Fig. 7:**
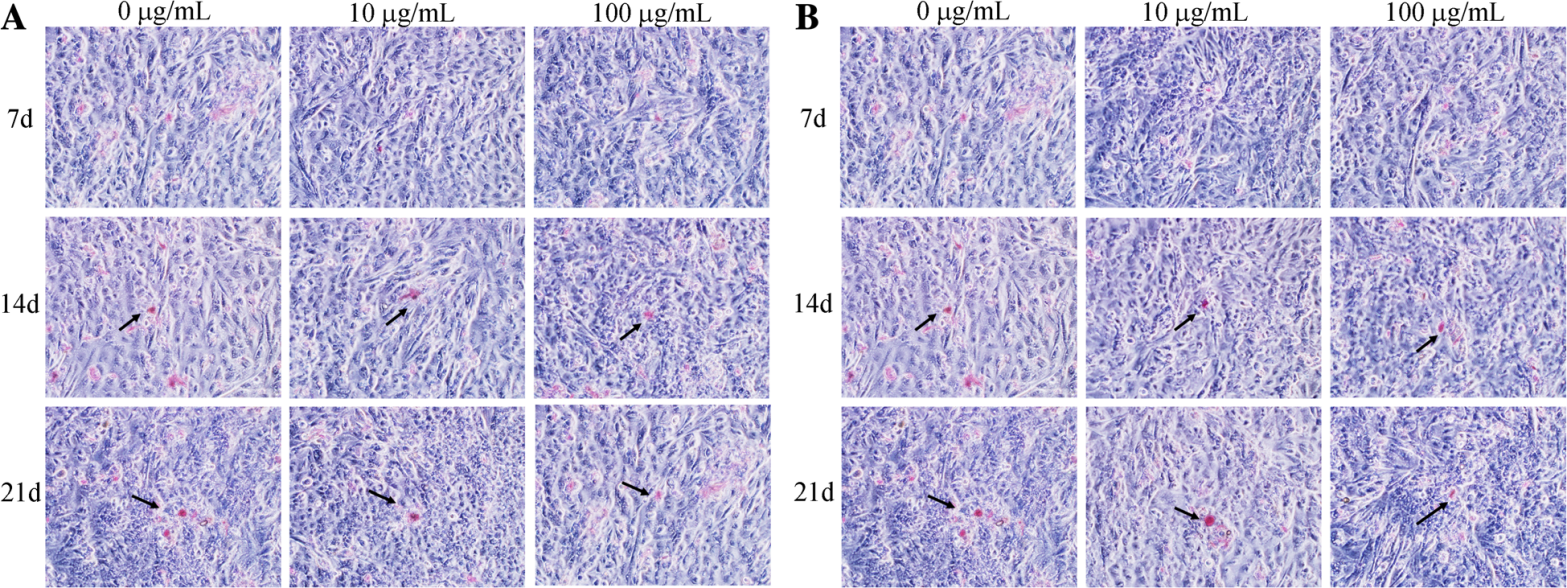
TiO_2_ and ZrO_2_ NP-induced mineralization effects in 3T3 cells. After the 3T3-E1 cells were differentiated using mineralized solution for 7 d, 14 d and 21 d, accompanied with TiO_2_ (**a**) and ZrO_2_ NPs (**b**) at various concentrations. The alizarin red staining was used to detect the mineralized nodule (black arrow)

Next, we detected the mineralization status of 3T3 cells by alizarin red staining and observed the formation of mineralized nodules under light microscopy (Fig. 7). Cells were stained after osteogenic induction for 7, 14, and 21 days in the presence of various concentrations of TiO_2_ and ZrO_2_ NPs. We found that mineralized nodules became visible after 14 and 21 days induction. There was no significant difference on mineralization after 14 and 21 days induction between the control group and TiO_2_ and ZrO_2_ NP treatment at 10 μg/mL. However, decrease of mineralization probably was observed after TiO_2_ and ZrO_2_ NP treatment at 100 μg/mL, due to the mineralized nodule that got smaller and blurry.

### TiO_2_ and ZrO_2_ NP-Induced Expression of Osteogenesis-Related Genes in 3T3 Cells

**Fig. 8:**
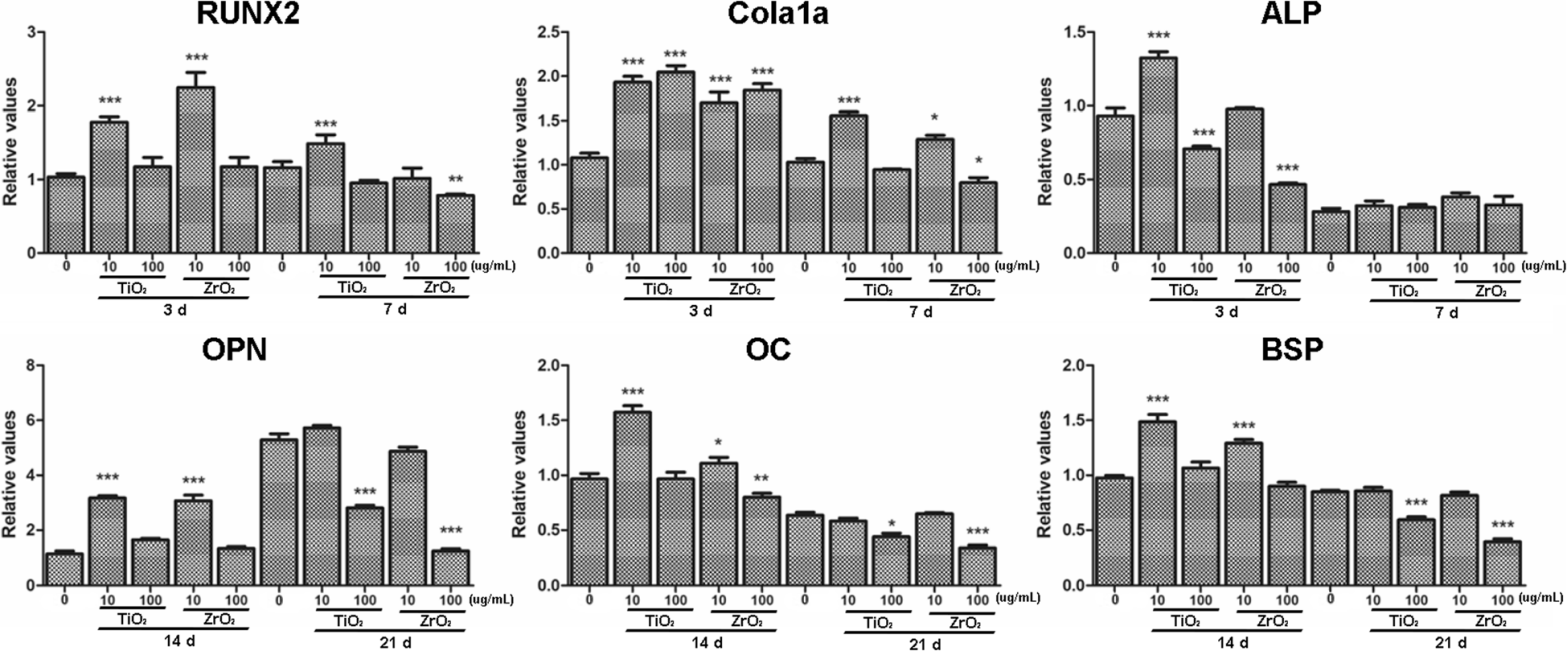
TiO_2_ and ZrO_2_ NP-induced osteogenesis-related genes changes in 3T3 cells. After the 3T3-E1 cells were differentiated using mineralized solution for 3, 7, 14, and 21 d, accompanied with TiO_2_ and ZrO_2_ NPs at various concentrations. The osteogenesis-related gene changes were detected using RT-PCR. The results represent the means ± SEM of three independent experiments. *p < 0.05; **p < 0.01; ***p < 0.001, compared with the control

In order to investigate the mechanism of TiO_2_ and ZrO_2_ NP-induced osteogenesis in 3T3 cells, we detected the levels of osteogenesis-related genes in 3T3 cells after TiO_2_ and ZrO_2_ NP treatment, including genes that preferentially upregulated during the early (*Runx2*, *Col1α1*, and *Alp*) and late (*Opn*, *Ocn*, and *Bsp*) phases of osteogenesis (Fig. 8). We found that 10 μg/mL of TiO_2_ and ZrO_2_ NPs induced the highest expression level of *Runx2* after 3 days of treatment, while at day 7, *Runx* decreased to the lowest level after ZrO_2_ NP treatment at 100 μg/mL. *Col1α1* increased after 10 μg/mL of TiO_2_ and ZrO_2_ NP treatment both at days 3 and 7, while for cells treated with 100 μg/mL of TiO_2_ and ZrO_2_ NPs, *Col1α1* first significantly upregulated at day 3 but decreased dramatically after 7 days. We also detected significant decrease of *Alp* expression after TiO_2_ and ZrO_2_ NP treatment at 100 μg/mL for 3 days.

For genes upregulated in the late phase of osteogenic induction, the expression levels of *Opn*, *Ocn*, and *Bsp* increased significantly after 10 μg/mL of TiO_2_ and ZrO_2_ NP treatment for 14 days, and *Opn* continuously upregulated to a higher level at day 21. These results suggested that compared with *Ocn* and *Bsp*, *Opn* was a later stage marker of TiO_2_ and ZrO_2_ NP-induced osteogenesis. Interestingly, 100 μg/mL of TiO_2_ and ZrO_2_ NPs failed to enhance the expression of *Opn*, *Ocn*, or *Bsp* at day 14; moreover, these genes showed significant downregulation at day 21.

## Discussion

ZrO_2_ NPs were important components in refractories, ceramics, and biomedical appliances, including implants, joint endoprostheses, and dental materials. Until now, TiO_2_ NPs as one of the other NPs with similar physicochemical properties, many studies have focused on its toxicological data. They found that TiO_2_ NPs could translocate into cells and showed potential cell damage due to different physicochemical characteristics [20, 21]. Meanwhile, the toxicological data for ZrO2 NPs was lacking. In our study, we regarded TiO_2_ NPs as the control group and explored the toxicological effects of TiO_2_ and ZrO_2_ NPs on 3T3-E1 cells. Physicochemical properties of NPs, especially size and morphology, have been known to effectively impact biosafety. Some studies have shown that nanoscaled particles were significantly more toxic than microscaled particles [22, 23]. In most cases, particle morphology was also reported to affect the toxicity [24–26]. In our study, we showed that TiO_2_ and ZrO_2_ NPs were rod-shaped spheres. Compared with previous reports [5, 27, 28], our TiO_2_ and ZrO_2_ NPs had a relatively weaker agglomeration effect in water where the particles enlarged to 81.2 and 93.1 nm in size, while we also could observe some microscale materials in culture medium after NP exposure with concentration-dependent manner, which confirm the agglomeration effect in this study even after using ultrasonic dispersion technology. However, the agglomeration effect could not inhibit the NP translocation into the cytoplasm, due to potent NPs were detected in intracellular vesicles. Organelles, like mitochondria, probably was one main target.

We have detected the viability of 3T3-E1 cells at various concentrations of TiO_2_ and ZrO_2_ NP treatment. Our results showed that 10 μg/mL of TiO_2_ and ZrO_2_ NPs is a biosafety concentration for 3T3-E1 cells. The cell viability decreased in time- and concentration-dependent manner, which implied that TiO_2_ and ZrO_2_ NPs were potentially cytotoxic after longer exposure of higher doses compared with other oxide metal nanoparticles, such as silicon dioxide and ZnO [4, 28, 29]. Moreover, ZrO_2_ NPs showed more potent toxic effects than TiO_2_ NPs in our study at high toxic concentrations.

Oxidative stress, a byproduct of outpaced ROS generation and decreased antioxidant factors, is known as one crucial factor in nanomaterial-induced cytotoxicity, and it is reported to trigger cell apoptosis through distinct mechanism [30, 31]. Furthermore, Kozelskaya et al. [12] observed that ZrO_2_ NPs induced the increase of membrane microviscosity, cell morphology changes, and surface cracks on the red blood cells due to the oxidative stress. In agreement with these studies, we detected the ROS levels in 3T3-E1 cells after TiO_2_ and ZrO_2_ NP treatment and found that TiO_2_ and ZrO_2_ NPs could induce significant ROS generation in concentration-dependent manners, and ZrO_2_ NPs induced more potent oxidative stress effects. Moreover, the elevated ROS levels could be eliminated by NAC which is a ROS scavenger. These results suggested the important role of ROS in TiO_2_ and ZrO_2_ NP-induced cell cytotoxicity.

Apoptosis is a type of cell death which clears the senescent and abnormal cells, so as to sustain the cell biological functions [32]. Some studies have reported that apoptosis was one of the main toxic responses after treating with oxide metal nanomaterials, such as TiO_2_, ZnO, Si, and Ag [33–36]. In our study, we found that TiO_2_ and ZrO_2_ NPs could induce apoptotic/necrotic body formation in 3T3-E1 cells in time/concentration-dependent manners, which was correlated with the decreased cell viability shown previously. Moreover, we found that when large parts of late apoptotic or necrotic cells were observed after ZrO_2_ NP treatment, the cell status for TiO_2_ NPs largely was early apoptosis. These phenomena applied that ZrO_2_ NPs induced more rapid and potent apoptosis effects. Similarly, other studies also showed that ZrO_2_ NPs induced significant apoptotic and necrotic processes in MSTO cells [4, 11].

The cytoskeleton metabolism is a dynamic biological process involving polymerization and depolymerization, which could sustain cell morphology and promote cell function. Some studies have shown that nanomaterials could affect the cell morphology and cytoskeleton system [37–39]. We found 3T3-E1 cells became smaller and rounded in the high-dose group of TiO_2_ and ZrO_2_ NPs (100 μg/mL), along with decreased cell area due to cytoskeleton disruptions. These findings were also supported by previous reports that ZrO_2_ NP treatment could induce cell morphology changes in MSTO cells at higher concentration [4]. Another study showed the disrupted blood cell morphology after ZrO_2_ NP treatment [12].

Alizarin red staining is a key indicator of osteogenic responses. In our study, no impact on osteogenic induction has been shown by TiO_2_ and ZrO_2_ NP treatment (10 μg/mL), except that cells treated with a cytotoxic dose of TiO_2_ and ZrO_2_ NPs (100 μg/mL) had a significant decrease of mineralized nodules due to the potential inhibition of osteoinductive properties. In addition, the expression level of osteogenesis-related genes was important biomarkers. Our results showed that lower concentration (10 μg/mL) of TiO_2_ and ZrO_2_ NPs promoted the expression of osteogenesis-related genes; however, TiO_2_ and ZrO_2_ NPs at high concentrations (100 μg/mL) could significantly inhibit gene expression for both early- and late phases of mineralization, indicating that TiO_2_ and ZrO_2_ NPs at high concentrations indeed inhibited osteoinductive properties. Other studies also obtained similar results; they claimed that TiO_2_ NPs inhibited the osteogenesis of osteoblasts in a size-dependent manner while potentially promoted osteoclastogenic process [33]. Sengstock et al. [40] found that sub-toxic concentrations of Ag NPs and Ag ions could significantly impair the osteogenic differentiation of human mesenchymal stem cells. More ongoing or newly initiated researches are focused on developing nanoparticles with acceptable biosafety and osteogenic potential to promote osseointegration for in vivo application [18, 41].

## Conclusion

In conclusion, our data indicated that ZrO_2_ NPs were nanoparticles with good biocompatibility, just like TiO_2_ NPs, while they could induce toxic effects at high toxic concentrations on 3T3-E1 cells. ROS played a key role on TiO_2_ and ZrO_2_ NP-induced cytotoxicity, including cell viability, apoptosis and necrosis, and changes in cell morphology. Moreover, TiO_2_ and ZrO2 NPs at high concentrations showed inhibitory effects on osteogenic differentiation of 3T3-E1 cells. Our findings could provide deep insights into the biocompatibility and potential application of ZrO2 NPs.

## Abbreviations

Ag: Silver
ALP: Alkaline phosphatase
Col1α1: Collagen 1α1
DLS: Dynamic light scattering
FBS: Fetal bovine serum
NAC: *N*-acetyl-l-cysteine
NPs: Nanoparticles
OC: Osteocalcin
OD: Optical density
OPN: Osteopontin
PBS: Phosphate-buffered saline solution
ROS: Reactive oxygen species
RUNX2: Runt-related transcription factor 2
Si: Silica
TEM: Transmission electron microscope
TiO_2_: Titanium dioxide
ZrO_2_: Zirconia
α-MEM: Minimum essential medium-alpha
